# Proteogenomics Uncovers Critical Elements of Host Response in Bovine Soft Palate Epithelial Cells Following In Vitro Infection with Foot-And-Mouth Disease Virus

**DOI:** 10.3390/v11010053

**Published:** 2019-01-12

**Authors:** Florian Pfaff, Sara Hägglund, Martina Zoli, Sandra Blaise-Boisseau, Eve Laloy, Susanne Koethe, Daniela Zühlke, Katharina Riedel, Stephan Zientara, Labib Bakkali-Kassimi, Jean-François Valarcher, Dirk Höper, Martin Beer, Michael Eschbaumer

**Affiliations:** 1 Institute of Diagnostic Virology, Friedrich-Loeffler-Institut, Federal Research Institute for Animal Health, 17493 Greifswald, Germany; Florian.Pfaff@fli.de (F.P.); martina.zoli2811@gmail.com (M.Z.); Susanne.Koethe@fli.de (S.K.); Dirk.Hoeper@fli.de (D.H.); Martin.Beer@fli.de (M.B.); 2 Swedish University of Agricultural Sciences, Host-pathogen interaction group, Division of Ruminant Medicine, 75007 Uppsala, Sweden; sara.hagglund@slu.se (S.H.); jean-francois.valarcher@slu.se (J.-F.V.); 3 Laboratoire de Santé Animale de Maisons-Alfort, UMR 1161 virologie, INRA, Ecole Nationale Vétérinaire d’Alfort, ANSES, Université Paris-Est, 94700 Maisons-Alfort, France; sandra.blaise-boisseau@anses.fr (S.B.-B.); eve.laloy@vet-alfort.fr (E.L.); stephan.zientara@vet-alfort.fr (S.Z.); Labib.BAKKALI-KASSIMI@anses.fr (L.B.-K.); 4 Biopôle EnvA, Ecole Nationale Vétérinaire d’Alfort, Université Paris-Est, 94700 Maisons-Alfort, France; 5 Institute of Microbiology, Department for Microbial Physiology and Molecular Biology, University of Greifswald, 17489 Greifswald, Germany; daniela.zuehlke@uni-greifswald.de (D.Z.); riedela@uni-greifswald.de (K.R.)

**Keywords:** foot-and-mouth disease virus (FMDV), bovine soft palate, nasopharynx, transcriptomics, proteomics, bioinformatics, virus-host interaction, innate immune system, interferon-stimulated genes (ISG)

## Abstract

Foot-and-mouth disease (FMD) is the most devastating disease of cloven-hoofed livestock, with a crippling economic burden in endemic areas and immense costs associated with outbreaks in free countries. Foot-and-mouth disease virus (FMDV), a picornavirus, will spread rapidly in naïve populations, reaching morbidity rates of up to 100% in cattle. Even after recovery, over 50% of cattle remain subclinically infected and infectious virus can be recovered from the nasopharynx. The pathogen and host factors that contribute to FMDV persistence are currently not understood. Using for the first time primary bovine soft palate multilayers in combination with proteogenomics, we analyzed the transcriptional responses during acute and persistent FMDV infection. During the acute phase viral RNA and protein was detectable in large quantities and in response hundreds of interferon-stimulated genes (ISG) were overexpressed, mediating antiviral activity and apoptosis. Although the number of pro-apoptotic ISGs and the extent of their regulation decreased during persistence, some ISGs with antiviral activity were still highly expressed at that stage. This indicates a long-lasting but ultimately ineffective stimulation of ISGs during FMDV persistence. Furthermore, downregulation of relevant genes suggests an interference with the extracellular matrix that may contribute to the skewed virus-host equilibrium in soft palate epithelial cells.

## 1. Introduction

Foot-and-mouth disease (FMD) is an acute and severe systemic vesicular disease of cloven-hoofed animals (*Artiodactyla*) with tremendous economic impact. The last FMD epizootic in the European Union in the United Kingdom, Ireland, France and the Netherlands in 2001 culminated in the slaughter of more than 6.5 million animals and an economic toll of over €5 billion [1]. The etiological agent is foot-and-mouth disease virus (FMDV), the type species of the genus *Aphthovirus* in the family *Picornaviridae* [2]. FMDV particles comprise a non-enveloped icosahedral capsid that surrounds a single-stranded positive-sense RNA genome with an approximate length of 8.4 kilobases [3]. FMD mainly affects livestock such as cattle, buffalo, pigs, goats, and sheep, but can also be transmitted to deer and wild boar. It is endemic in wild buffalo in Southern Africa [4,5]. Although more than 70 species are known to be susceptible to FMDV, its primary host seem to be buffalo and cattle, in which the disease causes very high morbidity, but only low mortality in adults [6]. During the onset of acute infection, cattle are highly febrile and small vesicles develop on the mucosal membranes of the muzzle, lips, and oral cavity, as well as on the coronary band and interdigital space, and the teats of the udder.

Usually, cattle clinically recover within 2–3 weeks if no secondary infection occurs. At this time, many have completely cleared the virus, however, about 50% of animals may remain subclinically infected for up to three years, depending on the species [7]. The World Organisation for Animal Health (OIE) defines an animal from which infectious FMDV can be recovered by probang sampling later than 28 days post infection (dpi) as persistently infected or a so-called “carrier” [7]. The fear of contagion from carrier animals has severe consequences for trade in live animals and animal products [8]. In vivo studies have shown that in cattle more than 50% of animals will become persistently infected [9], even if they had been vaccinated against FMDV and did not develop clinical disease [10,11,12]. The exact anatomical sites of persistence are still debated, but different tissues of the upper respiratory tract including the nasopharynx have been suggested. A recent study of the tissue-specific localization of FMDV in persistently infected steers identified the contiguous epithelia of the dorsal soft palate (SP) and the dorsal nasopharynx as the most likely sites of persistence [10]. Evidence for FMDV persistence in lymph nodes and germinal centers was also put forward although no viral replication could be detected [13].

The cellular factors that promote establishment of FMDV persistence and the viral strategies of immune avoidance in the bovine nasopharynx remain currently poorly understood, making it impossible to predict which animals will develop into carriers and which will clear the virus. Previous in vitro work that aimed to decipher cellular responses during persistent FMDV infection hardly reflected persistence in vivo as the used models were either based on immortalized non-bovine cell lines, such as hamster kidney (BHK) cells [14], or bovine cell lines, e.g., bovine kidney (MDBK, EBK) cells [15,16], that are not from the primary site of persistence. O’Donnell et al. [17] used a persistently infected bovine pharynx cell line to examine the gene expression of selected cellular cytokines by RT-qPCR, showing differences in the expression of key antiviral cytokines between acute and persistent infection.

The rationale of this study was therefore to use a novel air–liquid interphase cell culture model based on primary SP cells from cattle [18] that closely resembles the situation in vivo, together with an proteogenomics approach that combined transcriptomics by high-throughput sequencing, RT-qPCR, proteomics and bioinformatics. Our results provide detailed insights into the transcriptional responses of the SP in reaction to acute FMDV infection and reveal long-lasting changes of gene activation throughout persistence. The identified pathways and genes may give rise to further investigations leading to early detection of persistence in cattle and novel vaccines that prevent the carrier state.

## 2. Materials and Methods

A schematic visualization of the sample processing and analysis workflow can be found in the Supplementary Figure S1.

### 2.1. Ethics Statement

The tissues used for the study were collected from animals slaughtered for food production. The animals were being processed as part of the normal work of the abattoir, therefore no ethics approval was required.

### 2.2. Bovine Epithelial Cultures from Soft Palate

Multilayers of bovine dorsal soft palate cells were propagated at the air–liquid interface for 5 weeks before FMDV infection, as described previously [18]. Briefly, bovine dorsal soft palate tissue, collected immediately after slaughter, was dissected and digested at 4 °C overnight in incubation medium supplemented with protease XIV (Sigma-Aldrich, St. Louis, MO, USA). Epithelial cells were thereafter scraped off the underlying tissue, filtered, and incubated in cell culture flasks for 4 h at 37 °C and 5% CO_2_. Cells that did not adhere to the plastic were centrifuged at 200× *g* for 10 min at room temperature, frozen, thawed, and propagated for three to five passages in cell culture flasks before being seeded in 12 mm diameter Corning® Transwell-COL collagen-coated PTFE membrane inserts with 3.0 μm pores (Sigma-Aldrich). The cell culture medium was removed from the upper compartment after five days of culture and changed in the lower compartment every two or three days. The average number of cells that constituted the upper layer of the multilayer was estimated at 750,000.

### 2.3. Experimental Design and FMDV Infection

After 5 weeks of culture on inserts without passage, cells were infected with a twice-plaque-purified viral clone (FMDV O Clone 2.2, “Cl 2.2”) derived from the O/FRA/1/2001 strain that was further propagated on BHK-21 cells (four passages) [16], or negative cell lysate, as described previously [18]. Two experiments (experiment 1 and 2) were performed with SP cells that originated from two different animals (a male and female, respectively) and that were infected at a multiplicity of infection (MOI) of 0.01 (compare Supplementary Table S1). Briefly, the inserts were incubated for one hour with 500 µL of clarified cell lysate from infected or uninfected cell cultures. Following infection and thereafter at a maximum interval of 3 days, the upper compartments were washed with 500 µL cell culture medium containing 10% FCS and, similarly, but only from 2 dpi, the medium in the lower compartments was changed. For each experiment, at days 0, 1 and 28, SP cells from 2 inserts were lysed with 750 μL of TRIzol Reagent (Life Technologies, Carlsbad, CA, USA) and frozen separately at −80 °C for transcriptomic and proteomic analyses.

### 2.4. RNA and Protein Isolation

For isolation of high-quality total RNA and proteins, 150 µL of trichlormethane (Carl Roth, Karlsruhe, Germany) was added to the lysed cells in 750 μL TRIzol Reagent and the mixture was centrifuged in order to separate the RNA-containing aqueous phase from the DNA- and protein-containing organic phase. The aqueous phase was then mixed with an equal amount of 100% ethanol (Carl Roth) and total RNA was extracted using the RNeasy Mini Kit (Qiagen, Hilden, Germany) with on-column DNase digestion with the RNase-Free DNase Set (Qiagen), following the manufacturer’s instructions. The quantity and quality of total RNA was subsequently analyzed using a NanoDrop 1000 spectrophotometer (Peqlab, Erlangen, Germany) and RNA 6000 Pico chips on an Agilent 2100 Bioanalyzer (Agilent Technologies, Böblingen, Germany). All samples were checked for contamination using the 260/280 and 260/230 nm ratios, as well as for RNA degradation using the RNA Integrity Number (RIN).

Polyadenylated mRNA was subsequently isolated from 1–3 µg of high-quality total RNA using the Dynabeads mRNA DIRECT Micro kit (Invitrogen, Carlsbad, CA, USA) following the manufacturer’s instructions. Prior to isolation, the ERCC ExFold RNA Spike-In mix 1 (Invitrogen) was supplemented and used as an internal control for all following steps. The quality of the mRNA and the extent of ribosomal RNA contamination was assessed using RNA 6000 Pico chips on the Agilent 2100 Bioanalyzer (Agilent Technologies).

Proteins were extracted from the organic TRIzol phase following the manufacturer’s instructions. Briefly, 0.3 mL of 100% ethanol (Carl Roth) were added and DNA was pelleted by centrifugation. To the supernatant, 1.5 mL of isopropanol (Carl Roth) were added and proteins were pelleted by centrifugation. Protein pellets were washed three times with 0.3 M guanidine hydrochloride (Carl Roth) in 95% ethanol (Carl Roth). After a final washing step using 95% ethanol, proteins were air-dried and the pellet was resuspended in freshly prepared 1% SDS (Carl Roth) by ultrasonication. Quality and quantity of the isolated proteins were checked using a 12% polyacrylamide gel (SDS-PAGE) and a colorimetric bicinchoninic acid (BCA) assay.

### 2.5. Library Preparation and Sequencing

For preparation of whole-transcriptome libraries the Ion Total RNA-Seq Kit v2 (Life Technologies) was used, following the manufacturer’s instructions. Briefly, between 1 and 100 ng of the mRNA containing the ERCC spike-in control was treated with RNase III at 37 °C for 10 min. The fragmented mRNA was subsequently purified using the Magnetic Bead Cleanup Module (Life Technologies) and the resulting size distribution was assessed with the Agilent 2100 Bioanalyzer as described above. After hybridization and ligation of appropriate adapters, the fragmented mRNA was reverse transcribed into cDNA using SuperScript III enzyme. The cDNA was purified as described above and amplified for 14 cycles using Platinum PCR SuperMix High Fidelity along with appropriate primers for the generation of barcoded libraries. The resulting libraries were again purified using the method described above and the size distribution was assessed using the Agilent 2100 Bioanalyzer together with the DNA 7500 kit and chip (Agilent Technologies). All libraries were subsequently quantified using the KAPA Library Quantification Kit Ion Torrent (Kapa Biosystems, Wilmington, MA, USA) on a CFX96 Real-Time PCR Detection System (Bio-Rad Laboratories, München, Germany) and pooled at an equimolar ratio. For sequencing an Ion S5XL sequencing system (Life Technologies) along with the Ion 540 OT2 and Chip kit (Life Technologies) for the generation of up to 200 bp reads was used. Each library was sequenced in at least two independent sequencing runs.

### 2.6. Statistical Analysis of Differential Gene Expression

In order to detect problems and biases during mRNA isolation and library preparation, we used the ERCC_Analysis Plugin (version 5.8.0.1) provided in the Torrent Suite software (version 5.8.0). Only forward strand reads were selected for mapping and the minimum transcript count was set to 50. The raw reads from each sequencing library were quality checked using FastQC (version 0.11.7; Babraham Institute) with a focus on the read length distribution and adapter contamination. In order to quantify the expression of known bovine transcripts in these datasets we used Salmon (version 0.9.1) that uses a lightweight alignment method (quasi-mapping) for rapid transcript abundance estimation [19]. Briefly, we obtained the transcript reference of cattle (GCF000003055.6 Bos Taurus UMD 3.1.1) from NCBI and selected only transcripts that were featured as mRNAs, non-coding RNAs and miscellaneous RNAs (for details see Supplementary Table S2). A Salmon index was created using the option for a perfect hash, rather than a dense hash. Each sequencing library was then used as input for the major Salmon function “quant” using appropriate options for the library type (stranded single-end protocol with reads coming from the forward strand) and the mean read length. The number of bootstraps was set to 100 replicates. Subsequently a “tx2gene” table was prepared using the accessions of each RNA transcript from the aforementioned reference in the first column and the corresponding gene symbol in the second column (see also Supplementary Table S3). Using this table, the transcript abundancy datasets from Salmon “quant” were imported into R workspace (version 3.4.1; [20]) using the “tximport” package (version 1.6.0; [21]) as implemented in the Bioconductor library. For handling and manipulating R scripts, the software RStudio (version 1.0.153) was used. Technical replicates of samples (multiple sequencing of the same library from a single sample) were combined and the datasets were pre-filtered using only genes were more than four samples had raw gene counts greater than or equal to 100. In order to check the dataset for influence of treatment (infection, time and animal) and repeatability (replicates of same treatment) a principal components analysis (PCA) as well as a heatmap clustering was conducted using the regularized log transformed (rlog) read counts. DESeq2 (version 1.18.1; [22]) was then used to identify differentially expressed genes between the treatments based on the negative binomial distribution. The resulting *p*-values were adjusted with the Benjamini–Hochberg procedure and only genes with an adjusted *p*-value below 0.001 and an absolute fold change of >1 were considered significant. The logarithmic fold changes were further shrunken as recommended and described by Love et al. 2014 [22] in order to account for genes with low read counts. Significant differently expressed genes were annotated using the AnnotationDbi package (version 1.40.0) and used for further pathway enrichment analysis. Briefly, gene sets were analyzed using the “enrichPathway” function of the ReactomePA package (version 1.22.0; [23]) and the “enrichKEGG” function of the clusterProfiler package (version 3.6.0; [24]).

### 2.7. Quantitative Reverse Transcription PCR (RT-qPCR)

In order to confirm the results from the RNA sequencing experiment and to include samples from additional time points, a subset of six target genes (*ANKRD1*, *CASP7*, *IDO1*, *IFIH1*, *NCAM1*, *OAS2*) and two reference genes (*ACTB*, *GAPDH*) was selected for quantitative reverse transcription PCR (RT-qPCR) analysis. For each gene, two intron-spanning primer sets were designed using Primer3 (version 0.4.0; [25]) (for primer sequences see Supplementary Table S4). For RT-qPCR the QuantiTect Probe RT-PCR Kit (Qiagen) was used together with LightCycler 480 ResoLight Dye (Roche, Mannheim, Germany) according to the manufacturers’ instructions. The following temperature profile was used on a Bio-Rad CFX96: 50 °C for 30 min, 95 °C for 15 min and 45 cycles of 94 °C for 15 s and 60 °C for 1 min. After each cycle, the fluorescence in the SYBR channel was detected and threshold cycle (Ct) values were deduced after each run. For each newly designed primer set the PCR efficiency was determined using an appropriate dilution series from an independent bovine RNA control. Using the ∆∆Ct method, Ct values of target genes were first normalized by subtracting the Ct value of references genes (∆Ct = Ct_Target_ − Ct_Reference_). Mean and standard deviation of ∆Ct were calculated from two technical and two biological replicates for each treatment group. Subsequently, the ∆∆Ct was calculated by subtracting the ∆CT of the control group from the ∆Ct of the treatment group at different time points (∆∆Ct = ∆Ct_Control_ − ∆Ct_Treatment_) and used for calculation of the fold change of each gene (2^−∆∆CT^).

### 2.8. Protein Identification and Quantification, and Statistical Analysis of Differential Protein Expression

Thirty µg of the extracted proteins were separated by SDS-PAGE, stained with Colloidal Coomassie Brilliant Blue G-250 and afterwards lanes were cut into ten equidistant pieces. In-gel digestion using trypsin and purification of tryptic peptides using Ziptips (C18, Millipore) prior to MS analysis were done as described previously [26]. LC-MS/MS were conducted using an EASY-nLC II coupled to a LTQ Orbitrap-Velos mass spectrometer (Thermo Fisher Scientific, Waltham, MA, USA). Peptides were separated at a constant flow rate of 300 nL/min using a binary 76 min gradient from 5% B to 75% B 99.9% ACN, 0.1% acetic acid). Survey scans in the Orbitrap were recorded with a resolution of 60.000 in a m/z range of 300–1700. The 20 most intense peaks per scan cycle were selected for CID fragmentation in the LTQ. Ions with unknown charge state, as well as singly charged ions were excluded from fragmentation. Dynamic exclusion of precursor ions for 30 s was enabled. Internal calibration (lock mass 445.120025) was enabled as well. For protein identification, resulting spectra were searched against a database containing sequences of *Bos taurus* including reverse sequences and common laboratory contaminants (44,376 entries). Database searches using Sorcerer-SEQUEST (version v.27, rev.11; Sage-N Research, Inc., Milpitas, CA, USA) and Scaffold (version v.4.8.4; Proteome Software, Portland, OR, USA) were done as described earlier [27].

In order to identify differentially expressed proteins we imported the raw NSAF values into R workspace and created an ExpressionSet using the Biobase package (version 2.38.0; [28]). We then used the “Power Law Global Error Model” (PLGEM) as implemented in the “plgem” package (version 1.50.0; [29]) and first fitted it to the dataset using default settings and “FMDV infection” (samples from 24 h post infection (hpi) and 28 dpi) as fitting condition. After computation of observed and resampled signal-to-noise ratios, *p*-values for each detected protein were calculated.

### 2.9. Data Availability

The raw sequencing data along with deduced Salmon read count tables and substantial metadata are available at ArrayExpress (http://www.ebi.ac.uk/arrayexpress) under the accession number E-MTAB-7605. The mass spectrometry proteomics data have been deposited to the ProteomeXchange Consortium (http://www.proteomexchange.org) via the PRIDE [30] partner repository with the dataset identifier PXD012242

## 3. Results

### 3.1. A Cell Culture Model for FMDV Persistence

The establishment and full characterization of the primary SP cell culture model are presented by Hägglund et al. [18]. In summary, the primary SP cells formed into multilayers and showed typical features of stratified squamous epithelia, such as tight junctions and impermeability to cell culture media. The cultures were inoculated either with a viral clone derived from the O/FRA/1/2001 strain or negative cell lysate for control. Infection with FMDV at low MOI resulted in limited cytopathic effect, high viral loads and presence of detectable viral antigen. After 28 dpi, the multilayers were still intact, but FMDV antigen and genome remained detectable at very low levels and viable virus could be isolated—giving a clear indication of FMDV persistence.

### 3.2. RNA Sequencing and Exploratory Data Analysis

**Figure 1:**
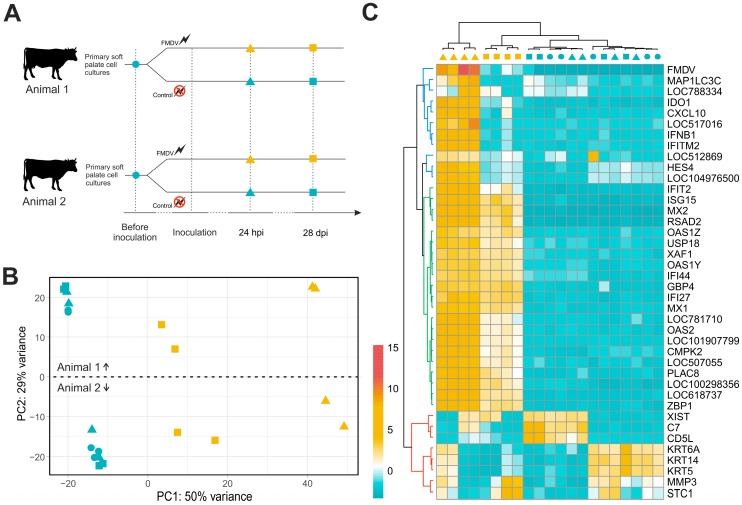
Experimental setup of RNA sequencing and exploratory data analysis. (**A**) Primary soft palate (SP) cell cultures were obtained from two animals. Baseline samples for RNA sequencing were collected immediately before inoculation (circle). Subsequently, the cell cultures were inoculated with foot-and-mouth disease virus (FMDV) (orange symbols) or mock-infected for use as controls (blue symbols). Cells were then harvested for sequencing at 24 h post infection (hpi) (triangles) or 28 days post infection (dpi) (squares), representing acute and persistent infection, respectively. (**B**) Principal components analysis based on normalized gene counts of the 1000 most variable genes. (**C**) The variance of normalized gene counts was calculated for each gene and the 45 genes with the highest variance were selected and visualized in a heat map. The color of the cells indicates the difference from the mean normalized gene count of the corresponding gene. Samples and genes were clustered according to these differences (trees). The transcript cluster highlighted in green is similarly activated by acute and persistent FMDV infection, while the blue cluster highlights transcripts that are only active during the acute phase of infection. The red cluster comprises transcripts whose abundance differs between donor animals used in this experiment.

The polyadenylated RNA fraction from 21 primary bovine SP cell culture samples was sequenced. From these, 10 and 11 samples originated from a female and male bovine, respectively. Cells for analysis were harvested immediately before inoculation (0 hpi), 24 hpi and 28 dpi (Figure 1A). For each time point (0 hpi, 24 hpi, 28 dpi) and treatment (FMDV, control), a minimum of four biological replicates (two from each animal) was sequenced. The number of reads for each sample ranged from 21.8 to 28.0 million, with an average of 24.7 million. In total, 518.4 million reads were included in the analysis (Supplementary Table S1).

These reads were assigned to a cattle transcript reference and the raw gene count data was transformed with respect to library size and transcript length using an appropriate model (Supplementary Figure S2). A principal components analysis (PCA) based on the normalized gene counts revealed that the samples from non-infected cultures remained closely together independent of the time in culture, while FMDV-infected samples showed a clear time-dependent grouping (Figure 1B). Accordingly, the first principal component (50% of variance) was assumed to represent the transcriptional differences between the samples caused by FMDV infection. Furthermore, transcriptional differences between both animals were clearly visible and represented by principal component 2 (29% of variance). The PCA showed no batch-to-batch effects and biological replicates from the same animal were grouped tightly.

Unsupervised cluster analysis of the 45 most variable gene transcripts clearly separated the FMDV-infected samples from the non-infected controls (Figure 1C). In detail, the infected samples showed a strong positive deviation from the per gene mean count (Figure 1C, green and blue transcript cluster). Interestingly, samples from the acute and persistent phases of infection were clearly separated, because the activation of the aforementioned genes was reduced at 28 dpi (Figure 1C, blue cluster). As in the PCA, the non-infected samples were divided by the differential expression of another set of transcripts (Figure 1C, red transcript cluster) and grouped according to the donor animal they originated from. In summary, the explorative data analysis showed that the FMDV infection has a clear effect on gene expression that is distinct from the animal-dependent effects.

### 3.3. Differential Expression during Acute and Persistent FMDV Infection and associated Pathways

**Figure 2:**
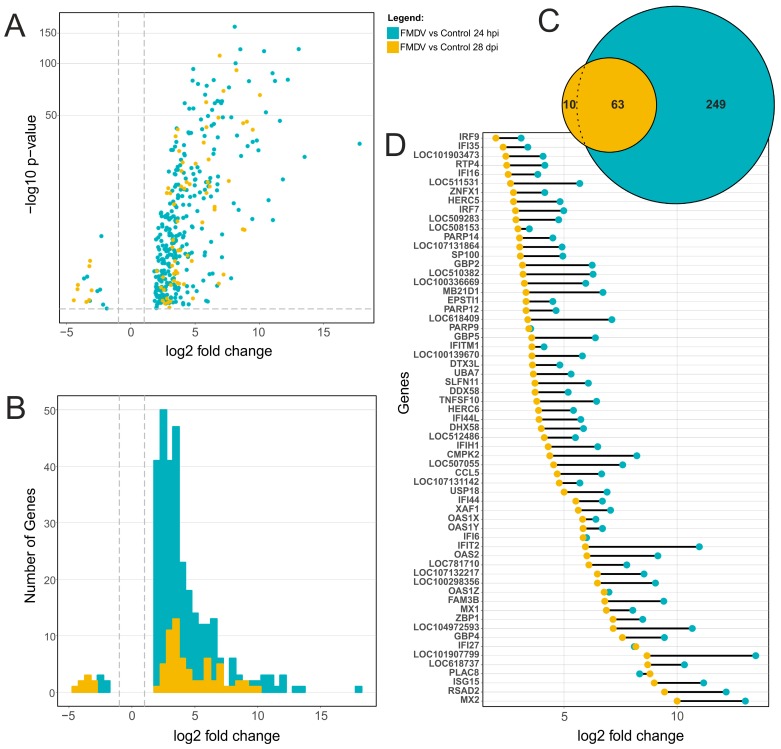
Differential gene expression of SP cells during FMDV infection. (**A**) Volcano plot showing the log2 fold change (*x*-axis) and the adjusted *p*-value (*y*-axis) for all differentially expressed genes during the acute (24 hpi, green) and persistent (28 dpi, orange) phase of FMDV infection. The log2 fold change and adjusted *p*-value are calculated relative to a non-infected control from the same time point. The grey dotted lines indicate the cutoff values: adjusted *p*-value < 0.001 and |log2 fold change| > 1. (**B**) The histogram summarizes the number of genes that have a certain log2 fold change. (**C**) The total number of differentially expressed genes is visualized in a Venn diagram. The overlap indicates the 63 genes that are differentially expressed during both phases of infection. (**D**) The log2 fold change of these 63 genes is compared for acute and persistent infection.

In order to confirm the trends from the explorative data analysis and to address the transcriptional changes during acute and persistent infection in more detail, a differential expression analysis was conducted. The transcriptional response of cells from bovine SP tissue to FMDV infection was assessed at 24 hpi and 28 dpi, in contrast to non-infected controls from the same time points (Figure 2). A total of 312 and 73 gene transcripts were differentially expressed at 24 hpi and 28 dpi, respectively. With 305/312 (97.7%) for 24 hpi and 65/73 (89%) for 28 dpi, the majority of differentially expressed transcripts were up-regulated, while only 8/312 (2.5%) and 8/73 (11%) were down-regulated (Figure 2A,B). From a total of 324 differentially expressed transcripts at both time points, 249 and 10 transcripts were solely regulated during either acute or persistent phase, respectively. The expression of 63 transcripts was significantly up-regulated during both stages of infection (Figure 2C). The log2 fold change of these transcripts was much lower at 28 dpi when compared to 24 hpi (Figure 2D), with highest differences observed for *IFIT2*, LOC101907799 and *CMPK2*. In contrast, the log2 fold change for *IFI27*, *PARP9*, *IFI6,* and *OAS1Z* was comparable or nearly equal during both phases of infection. *PLAC8* was slightly stronger regulated at 28 dpi than at 24 hpi. A full list of differentially expressed transcripts can be found in the supplementary material (Supplementary Table S5).

**Figure 3:**
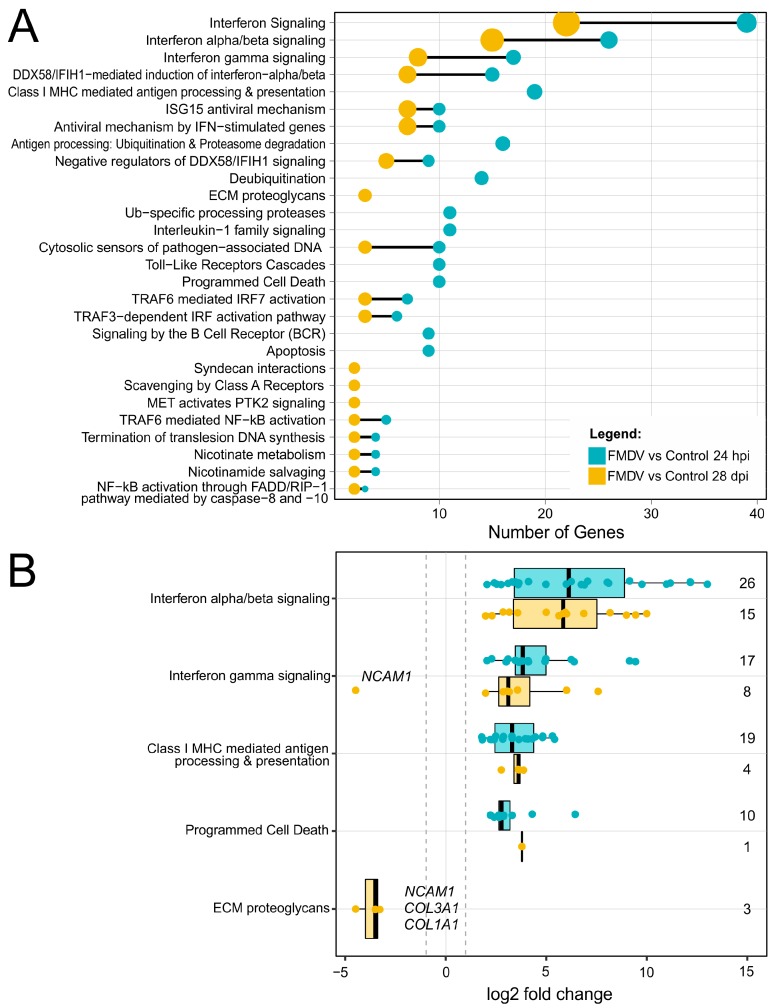
Pathway enrichment analyses of significantly differentially expressed genes during acute and persistent FMDV infection of bovine soft palate cells. (**A**) The number of genes that contribute to a significantly enriched Reactome pathway is shown for 24 hpi (blue dots) and 28 dpi (orange dots). Black lines connect pathways that are enriched in both datasets and the size of the circles represents the ratio of genes found for a certain pathway to the overall gene number. (**B**) The log2 fold change of genes that contribute to selected enriched Reactome pathways are highlighted (24 hpi—blue dots) and 28 dpi—orange dots). The grey dotted lines indicate a |log2 fold change| > 1. The numbers on the right correspond to the number of genes associated with each pathway.

The significantly differently expressed genes during acute and persistent infection were further matched to specific metabolic and signaling pathways using the Reactome database [31] (Figure 3). During both infection stages, most of the regulated genes are associated with interferon signaling, in particular interferon α, β and γ signaling (Figure 3A and Table 1). The induction of these interferons appears to be mediated by the DDX58/IFIH1 pathway. Accordingly, a number of interferon-stimulated genes (ISG), such as the ubiquitin-like family member *ISG15*, that induce strong antiviral mechanisms of the innate immune system are highly activated. During acute infection, transcripts involved in the interleukin-1 (*IL1*) and -10 signaling pathways as well as the programmed cell death (apoptosis) pathway were enriched. However, it is of note that the *IL1* and *IL10* genes were not significantly differently expressed. The MHC I antigen presentation pathway, a part of both the innate and adaptive immune system, was also enriched at 24 hpi. During persistence, most of the genes belonging to the interleukin-1, -10, apoptosis and MHC I antigen presentation pathways were not significantly differentially expressed in comparison to control samples.

All of the genes contributing to the aforementioned pathways were up-regulated during the acute and persistent phases of infection, with a single exception for the nuclear cell adhesion molecule 1 (*NCAM1*) that is related to interferon γ signaling and was down-regulated during persistence (Figure 3B, and Table 2). In addition to *NCAM1*, other proteins that are associated with extracellular membrane (ECM) pathways, such as type I and III collagens (*COL1A1* and *COL3A1*) were also down-regulated during persistence.

### 3.4. RT-qPCR and Quantitative Proteomics

**Figure 4:**
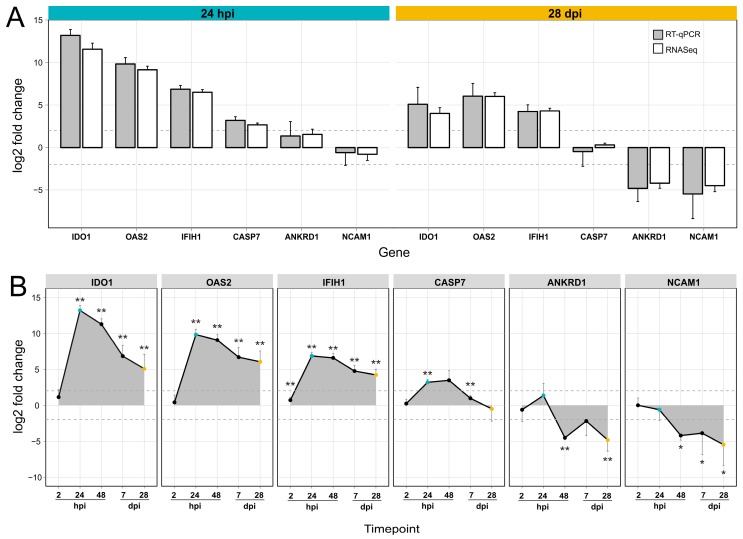
Expression analysis for selected genes by RT-qPCR at different time points. (**A**) Gene expression in FMDV infected soft palate cells compared to uninfected controls, at 24 hpi (blue panel) and 28 dpi (orange panel). The change in gene expression was analyzed for six selected target genes and two reference genes (not shown) using RT-qPCR. Log2 fold changes were calculated in comparison to a non-infected control from the same time points. Grey and white bars indicate the log2 fold change of gene expression using results from RT-qPCR and RNA sequencing (RNASeq), respectively. The grey dotted lines indicate a |log2 fold change| > 1. (**B**) The gene expression of the six selected genes was traced in a time course 2, 24, and 48 hpi; and 7 and 28 dpi. Statistically significant changes in comparison to control samples are highlighted with asterisks (*p*-value < 0.05: *; *p*-value < 0.01: **). Time points 24 hpi and 28 dpi are illustrated in blue and orange, respectively.

Results from the differential gene expression analysis based on RNA sequencing were confirmed using RT-qPCR. A panel of two reference (*ACTB*, *GAPDH*) and six target genes (*OAS2*, *IFIH1*, *NCAM1*, *ANKRD1*, *IDO1*, *CASP7*) were selected for analysis. These genes were chosen according to their apparent regulation: *OAS2*, *IFIH1* and *IDO1* were up-regulated during acute and persistent infection, while *NCAM1* and *ANKRD1* were only down-regulated during persistence. *CASP7* was only up-regulated during the acute phase of FMDV infection. The selected genes were also involved in relevant signaling pathways: *IFIH1* is part of the interferon induction cascade, while *OAS2* is an interferon-induced gene with antiviral activity. *CASP7* plays a key role in apoptosis and *IDO1* encodes a metabolically active protein that supports the immune system. *ANKRD1* and *NCAM1* were chosen as interesting target genes that were only regulated during persistence. The RT-qPCR analysis included samples from additional time points (2, 24, and 48 hpi; 7 and 28 dpi) for which log2 fold changes were then calculated using the delta-delta Ct method. For each time point and cell type four biological replicates (two replicates per animal) were used for analysis with the exception for 48 hpi, from which only two replicates derived from a single animal were available. The log2 fold-changes obtained by RT-qPCR of SP cells at the time points 24 hpi and 28 dpi confirm the results of the RNA sequencing (Figure 4A). All of the analyzed genes showed a time-dependent regulation during infection with FMDV (Figure 4B). The genes *IDO1*, *OAS2,* and *IFIH1* were strongly up-regulated from 2 to 24 hpi and were still up-regulated after 28 dpi. In contrast, *CASP7* was only up-regulated during acute infection. *ANKRD1* and *NCAM1* were down regulated as early as 48 hpi and throughout 28 dpi.

Additionally, a GeLC-MS/MS-based comparative proteome analysis was conducted to confirm the results of the RNA sequencing on the protein level, using two biological replicates for each time point and treatment from animal 2. Proteins with significantly different abundance in both experiments were identified and quantified by spectral-counting (Table 3). At the given thresholds, 11 and 8 proteins were differentially expressed when comparing FMDV-infected and control samples at 24 hpi and 28 dpi, respectively. Of these, 6 were differentially expressed at both time points. The Normalized Spectral Abundance Factor (NSAF), measured as the proportion of a single protein in relation to the detected proteins overall revealed an accumulation of the proteins *HERC6*, *IFI44*, *IFI44L*, *ISG15*, *MX1*, *MX2*, *OAS1X,* and *OAS1Y* in cells during persistent infection. In contrast, the proportion of *ATAD1*, *IFIT1,* and *IFIT2* to all other detected proteins was higher during acute than in persistent infection. FMDV polyprotein, *IFIT3* and *RSAD2* were not detected at the protein level at 28 dpi.

## 4. Discussion

The failure of the host response to clear virus from the nasopharynx is one of the most important features of FMDV infection, and has been studied previously using monolayers of primary cells and non-primary cell lines from hamsters (BHK-21) [14], swine (SK6) [32], and cattle (EBK, MDBK and pharynx cells) [15,16,17]. These cells needed regular cell culture passage and were, except for the pharynx cells, not from anatomical locations relevant to natural FMDV infection and persistence, which resulted in a suboptimal reflection of the in vivo situation.

In this study we used multilayers of primary bovine dorsal soft palate (SP) epithelial cells in an air–liquid interface cell culture model [18], as it has been shown that this tissue, along with the dorsal nasopharynx, is the most likely site of primary FMDV infection and persistence in cattle [10,33]. This model mimics several properties of the squamous epithelium that are observed in vivo and allows detailed studies of the target cells of primary FMDV infection. Specialized immune cells, such as B cells, T cells, and natural killer cells, which contribute to host responses in vivo, were depleted by the establishment of the cell culture in air [18], leading to improved standardization and a focus on the primary target cell. In vivo, neutralizing antibodies secreted by B cells are essential for resolving systemic FMDV infection, but have no effect on persistent infection in the nasopharynx [9]. Recent studies of gene expression in nasopharyngeal tissues of cattle have suggested an important role of cytotoxic T cells in the clearance of persistent FMDV infection [34,35]. The transcriptional changes analyzed in the present study, however, are limited to the response of SP epithelial cells themselves to infection with FMDV.

This response was analyzed using RNA sequencing, RT-qPCR and quantitative proteomics. Based on the results from exploratory RNA sequencing alone, infection with FMDV resulted in detectable changes in the transcriptome of the cells and the induced changes were distinct during the acute and persistent phases of infection. Despite that only a small proportion of cells were persistently infected, these influenced the transcriptome significantly. The non-infected controls did not show any comparable, time-dependent changes, indicating stable culture conditions without extensive cell differentiation or degradation. The observed changes induced by FMDV infection were also independent of the donor animals used for the preparation of the primary cell cultures, although transcriptional differences between the animals were detectable, e.g., sex-associated differences in *XIST* and keratin expression [36]. In summary, we observed genes that were highly regulated during both stages of infection (Figure 1C, green cluster), genes exclusively regulated during acute infection (Figure 1C, blue cluster) and animal-specific gene expression (Figure 1C, red cluster). During acute and persistent infection, comparably low numbers of genes were significantly differentially expressed and most of these were up-regulated. Interestingly, 63 genes were differentially expressed during both infection phases, indicating that FMDV infection induces long-lasting changes in the soft palate transcriptome. Furthermore, these genes were generally more up-regulated during acute infection. The transcriptional changes observed by RNA sequencing were supported at the protein level using quantitative proteomics. Although the number of detected differentially expressed proteins was much lower, it was confirmed that overexpression of ISGs leads to detectable levels of these proteins in the cells.

We found a strong activation of the innate immune response at 24 hpi that appears to be triggered by sensing of viral dsRNA over the cytosolic RNA sensors *IFIH1*/*MDA5* and *RIG-I*/*DDX58*, as the expression of MDA5 and RIG-I was significantly increased during acute infection. While RIG-I is sensing minus-strand RNA viruses by specific binding to 5′-triphosphate uncapped RNA genomes, MDA5 specifically senses plus-strand RNA viruses, such as FMDV, by binding to their dsRNA replication intermediates [37,38]. Although MDA5 is thought to be the main cellular detector of FMDV, it has been shown that RIG-I transcription is also elevated during FMDV infection [39]. After specific binding of MDA5 or RIG-I to their ligands they interact with TRIM25 and the mitochondrial protein MAVS that activate the transcription factors IRF3/IRF7 [40] as well as NF-κB [41], which is in accordance with the observed up-regulation of *IRF3*, *IRF7*, *NFKBIA*, *NFKBIB* and *TRIM25* (Table 1). Activated IRF3/IRF7 and NF-κB complexes thereafter induce the expression of type I/III interferons (IFNs) and proinflammatory cytokines, respectively [42]. We found that during acute infection especially IFN-β was expressed at elevated levels with significant overexpression of *IFNB1*, while IFN-α and IFN-λ were only expressed at very low levels (Supplementary Figure S3). However, we observed expression of several ISGs with antiviral activity, such as *IRF1*, *ISG15*, *MX1/2*, *OAS1/2,* and *RSAD2* (see Table 1). This indicates that while IFN-α transcription may be limited, IFN-β alone is able to induce a potent antiviral response in bovine SP cells. It is known that the viral leader proteinase L^pro^ of FMDV can inhibit IFN-β transcription and protein translation, thereby blocking the cells innate immune response [43]. However, at least in this model L^pro^ does not induce a full blocking of IFN-β transcription. A specific blocking of IFN-α transcription has only been shown for swine dendritic cell populations from blood and skin during acute FMDV infection [44]. The induction of IFN-β and associated ISGs was furthermore coincident with high viral genome copy numbers and presence of viral proteins (Table 3) as has been described before [45].

In contrast, no interferon expression was observed during the persistent phase of infection, although the aforementioned virus sensors MDA5/RIG-I and their associated transcription factor *IRF7* were still highly expressed (see *IFIH1* in Figure 3B). A possible factor reducing the transcription of interferons during persistence is the down-regulation of *ANKRD1*, as *ANKRD1* is directly involved in the signal transduction of *IRF3* and *IRF7* by binding IRF7/IRF3 complexes and thereby enhances the expression of type I/III interferons [46]. *ANKRD1* was initially observed in human dermal endothelial cells, where it is induced by inflammatory cytokines [47] and is substantially involved in the fibroblast mediated wound healing [48]. In agreement with our results, the expression of *ANKRD1* has previously been shown to be down-regulated in vivo during persistent FMDV infection of bovine nasopharyngeal tissue [35]. Gene silencing of *ANKRD1* in cells infected with herpes simplex virus resulted in increased viral load and reduced IFN-β (*IFNB1*) and IFN-λ (IL29) expression [46]. Therefore, the strong downregulation of *ANKRD1* as early as 48 hpi may be involved in the decrease of IFN-β expression during persistence.

Although no interferon expression was detectable at 28 dpi, the expression of many ISGs with known antiviral activity, such as *MX1/2* and *OAS1/2*, was significantly increased (see Figure 2D and Figure 3B). Furthermore, based on the proteome data, the translation of these genes was stable, as their encoded proteins accumulated in the cells (Table 3). This indicates long-lasting induction of ISGs during persistent infection, with viral protein below the detection limit and low FMDV genome copy numbers. A comparable pattern of highly expressed ISGs as observed during FMDV persistence (*ISG15*, *MX1*, *OAS1/2,* and *USP18*) has been identified in liver samples from chronic infections with hepatitis C virus (HCV) [49,50]. Interestingly, the elevated expression of *ISG15*/*USP18* during chronic HCV infection, as we also observed during FMDV persistence, correlated with decreased responses to IFN-α treatment. Furthermore, the strong expression of *OAS1/2*, *MX1/2* and *IRF7*, but not of *IRF3* at 28 dpi is in accordance with increased interferon receptor (*IFNR*) signaling [51,52]. Observations from persistent lymphocytic choriomeningitis virus (LCMV) infections revealed that persistence is driven by chronic *IFNR* signaling, characterized by similar ISG activation patterns as observed in the present study [52]. The blockade of *IFNR* by antibodies abolished the expression of *IL-10* and *PD-L1*, two immunosuppressive T-cell exhaustion factors expressed by dendritic cells, and ultimately led to clearance of persistent LCMV infection by activation of IFN-γ expressing CD4+ T cells [52]. In this study, we did not observe upregulation of *IL-10* or *PD-L1*, as no dendritic cells were present in the SP cultures; however, an overexpression of T-cell exhaustion factors was previously found in nasopharyngeal tissues of FMDV carrier animals [35] and therefore may play a role in FMDV persistence in vivo.

The immunosuppressive factors identified in that study also included transforming growth factor β (TGF-β). While an overexpression of TGF-β itself was not evident in our data, lymphocyte antigen 6 family member E (*LY6E*) was one of the two genes that were up-regulated during the persistent phase only. *LY6E*, an ISG, has been implicated in the TGF-β-mediated escape from immune surveillance in many forms of cancer [53]. *LY6E* has been previously shown to promote viral infection [54] and it is essential for clathrin-mediated endocytosis of virus particles [55], a pathway that is also used by FMDV [56].

During acute infection, the interferon-mediated induction of ISGs appears to trigger apoptosis, as indicated by pathway analysis. Interestingly, genes associated with apoptosis, such as *CASP7*, were only expressed during acute infection and their expression waned with increasing time post infection (Figure 3B). The apparent absence of apoptotic processes during persistent infection is in accordance with recent findings that indicated an inhibition of apoptotic pathways in nasopharyngeal tissues from FMDV carrier animals [34,35]. Furthermore, we observed a specific down-regulation of pro-apoptotic genes during persistence, such as *ALDH1A2*, *ANKRD1,* and *SFRP2* (Table 2). Similarly, overexpression of fructose-bisphosphatase 1, the other gene that was only up-regulated during the persistent phase, inhibits many forms of apoptosis by increasing total cellular glutathione [57].

Another interesting observation was the downregulation of *NCAM1* (CD56), a member of the immunoglobulin superfamily, as early as 48 hpi and its further decrease until 28 dpi. Besides its role as a differentiation marker for natural killer cells, *NCAM1* is involved in cell binding and migration [58]. Downregulation of *NCAM1* is associated with decreased cell adhesion capacity, enhanced tumor cell invasiveness and is triggered in other viral infections [59]. The expression of *HTRA3*, a serine protease involved in remodeling of the extracellular matrix (ECM), as well as the expression of the ECM components *COL1A1* and *COL3A1* were significantly down-regulated during persistent infection. The role of these changes during FMDV persistence is currently unknown but lends itself to some speculation. The interaction of cells with the ECM plays a key role in epithelial maturation [60], which in turn is critical for the life cycle of some persistent viruses such as papillomaviruses [61]. Even though FMDV and papillomaviruses are biologically very different, certain features of FMDV persistence in vivo are conspicuously similar to what is observed in papillomavirus infection—particularly the difference between the distribution of viral genome, which is concentrated in the basal stratum germinativum [62], and viral antigen, which is concentrated in the superficial layers of the soft palate epithelium [63]. This segregation helps papillomaviruses evade the immune system as high levels of viral replication and protein synthesis occur only in terminally differentiated cells that are not subject to immune surveillance [64]. Whether a similar mechanism is involved in the maintenance of FMDV persistence remains to be investigated. However, it is difficult to faithfully recreate the complex epithelial structure and ECM interactions in vitro and investigations of the role of the ECM in persistent FMDV infection have to be performed with ex vivo tissue samples.

## 5. Conclusions

In conclusion, our study independently confirms earlier findings of a polygenic inhibition of apoptosis during persistent FMDV infection, which has been put forward as one of the principal mechanisms for the maintenance of persistence [34,35]. Another proposed mechanism, Th2 polarization and T-cell exhaustion, was not directly represented in our data, because the SP culture model does not include specialized immune cells. Nevertheless, we demonstrated the utility of state-of-the-art proteogenomics for the analysis of transcriptional signatures of acute and persistent FMDV infection in a near-natural in vitro system. We will proceed to apply this technology to tissue samples collected from carrier animals to obtain the first comprehensive picture of the transcriptomic and proteomic alterations associated with FMDV persistence in the natural host. Unraveling the cellular mechanisms of FMDV persistence may ultimately give rise to improved diagnostics and prevention of the FMDV carrier state.

## Figures and Tables

**Table 1:** Selected enriched pathways and corresponding genes during acute and persistent FMDV infection.

Metabolic Complex	Pathway	24 hpi	28 dpi
Innate immune system	DDX58/IFIH1-mediated induction of interferon-alpha/beta	*DDX58, DHX58, HERC5, IFIH1, IFNB1, IRF1, IRF3, IRF7, ISG15, NFKBIA, NFKBIB, NLRC5, TNFAIP3, TRIM25, UBA7*	*DDX58, DHX58, HERC5, IFIH1, IRF7, ISG15, UBA7*
Cytokine signaling in immune system	Interferon alpha, beta signaling	*ADAR, GBP2, IFI27, IFI35, IFI6, IFIT2, IFIT3, IFITM1, IFITM2, IFNB1, IRF1, IRF3, IRF5, IRF7, IRF9, ISG15, MX1, MX2, OAS2, PSMB8, RNASEL, RSAD2, SOCS1, STAT2, USP18, XAF1*	*GBP2, IFI27, IFI35, IFI6, IFIT2, IFITM1, IRF7, IRF9, ISG15, MX1, MX2, OAS2, RSAD2, USP18, XAF1*
	Interferon gamma signaling	*GBP2, IFI27, IFI35, IFI6, IFIT2, IFITM1, IRF7, IRF9, ISG15, MX1, MX2, OAS2, RSAD2, USP18, XAF1*	*GBP2, GBP4, GBP5, IRF7, IRF9, NCAM1, OAS2, SP100*
	Interleukin-1 family signaling	*IL18, IL18BP, MAP3K8, NFKBIA, NFKBIB, PELI1, PSMB10, PSMB8, PSMB9, PSME2, PSMF1*	
	Interleukin-10 signaling	*CCL2, CCL5, CXCL10, CXCL2, CXCL8, IL18, IL6*	*CCL5*
	Antiviral mechanism by IFN-stimulated genes/ISG15 antiviral mechanism	*DDX58, EIF2AK2, HERC5, IRF3, ISG15, MX1, MX2, TRIM25, UBA7, USP18*	*DDX58, HERC5, ISG15, MX1, MX2, UBA7, USP18*
Adaptive immune system	Class I MHC mediated antigen processing and presentation	*AREL1, CTSS, DTX3L, ERAP2, HECTD2, HERC5, HERC6, PSMB10, PSMB8, PSMB9, PSME2, PSMF1, RBCK1, RNF114, RNF19B, SOCS1, TAP1, TRIM21, UBA7*	*DTX3L, HERC5, HERC6, UBA7*
	Activation of NF-kappaB in B cells	*NFKBIA, NFKBIB, NFKBIE, PSMB10, PSMB8, PSMB9, PSME2, PSMF1*	
Programmed cell death	Programmed cell death	*CASP7, CFLAR, PMAIP1, PSMB10, PSMB8, PSMB9, PSME2, PSMF1, RIPK3, TNFSF10*	*TNFSF10*

**Table 2:** Genes that are differentially expressed only during the persistent phase of FMDV infection.

Gene	Description	LFC^†^	Adjusted *p*-value	Enriched DAVID Terms
*NCAM1*	neural cell adhesion molecule 1	−4.48	3.35 × 10^−4^	signal peptide, secreted
*ANKRD1*	ankyrin repeat domain 1	−4.19	6.61 × 10^−5^	positive regulation of apoptotic process
*SFRP2*	secreted frizzled related protein 2	−4.16	3.31 × 10^−5^	signal peptide, secreted, positive regulation of apoptotic process
*COL1A1*	collagen type I alpha 1 chain	−3.50	4.16 × 10^−4^	signal peptide, secreted
*COL3A1*	collagen type III alpha 1 chain	−3.28	3.23 × 10^−4^	signal peptide, secreted
*MYLK*	myosin light chain kinase	−3.26	9.61 × 10^−7^	
*HTRA3*	HtrA serine peptidase 3	−3.21	2.93 × 10^−7^	signal peptide, secreted
*ALDH1A2*	aldehyde dehydrogenase 1 family member A2	−3.09	7.83 × 10^−5^	positive regulation of apoptotic process
*LY6E*	lymphocyte antigen 6 family member E	3.45	2.59 × 10^−6^	signal peptide
*FBP1*	fructose-bisphosphatase 1	4.09	2.94 × 10^−5^	

^†^: log2 fold change.

**Table 3:** Proteins with significantly different abundance at 24 hpi or 28 dpi. Only proteins that were also differentially expressed in the RNA sequencing experiment at the given time point are shown.

Protein	Description	24 hpi Mean NSAF	28 dpi Mean NSAF	NSAF_28 dpi_/NSAF_24 hpi_
*ATAD1*	ATPase Family, AAA Domain Containing 1	2.50 × 10^−4^ **	1.56 × 10^−4^	0.6
*CAG23917.14*	FMDV polyprotein	3.16 × 10^−4^ **	0 ^†^	n.a.
*HERC6*	HECT And RLD Domain Containing E3 Ubiquitin Protein Ligase Family Member 6	2.46 × 10^−5^	2.74 × 10^−4^ **	11.2
*IFI44*	Interferon Induced Protein 44	1.12 × 10^−4^ *	4.58 × 10^−4^ **	4.1
*IFI44L*	Interferon Induced Protein 44 like	8.32 × 10^−5^ *	6.98 × 10^−4^ **	8.4
*IFIT1*	Interferon-Induced Protein with Tetratricopeptide Repeats 1	5.41 × 10^−4^ **	7.62 × 10^−5^ *	0.1
*IFIT2*	Interferon Induced Protein With Tetratricopeptide Repeats 2	2.31 × 10^−4^ **	9.34 × 10^−5^ *	0.4
*IFIT3*	Interferon Induced Protein With Tetratricopeptide Repeats 3	3.78 × 10^−4^ **	0 ^†^	n.a.
*ISG15*	ISG15 Ubiquitin-Like Modifier	3.53 × 10^−3^ **	7.52 × 10^−3^ **	2.1
*MX1*	MX Dynamin Like GTPase 1	8.42 × 10^−4^ **	2.27 × 10^−3^ **	2.7
*MX2*	MX Dynamin Like GTPase 2	2.44 × 10^−4^ **	5.04 × 10^−4^ **	2.1
*OAS1X*	2′-5′-Oligoadenylate Synthetase 1 X	2.47 × 10^−4^ **	4.85 × 10^−4^ **	2.0
*OAS1Y*	2′-5′-Oligoadenylate Synthetase 1 Y	2.31 × 10^−4^ **	3.68 × 10^−4^ **	1.6
*RSAD2*	Radical S-Adenosyl Methionine Domain Containing 2	5.69 × 10^−4^ **	0 ^†^	n.a.

*: *p*-value < 0.01; **: *p*-value < 0.001; ^†^: not significant; n.a. not applicable.

## Supplementary Materials

The following are available online at http://www.mdpi.com/1999-4915/11/1/53/s1, Figure S1: Schematic visualization of the proteogenomic workflow, Figure S2: Comparison of different transformation methods for the raw read counts per gene, Figure S3: Comparison of interferon expression, Table S1: Summary of primary soft palate cell culture samples, Table S2: Summary of excluded and included RNA reference transcripts, Table S3: Replaced gene aliases of the *Bos taurus* reference genome, Table S4: Sequences of primers used for the RT-qPCR analysis, Table S5: Differentially expressed genes, File S1: Raw read counts for all significantly differently expressed genes.
