# Supplementary material for: Proteogenomics Uncovers Critical Elements of Host Response in Bovine Soft Palate Epithelial Cells Following In Vitro Infection with Foot-And-Mouth Disease Virus
Source: Viruses. 2019 Jan 12;11(1):53. doi: 10.3390/v11010053 (PMC6356718; doi:10.3390/v11010053)
Supplement: Supplementary file 1 [file viruses-11-00053-s001.zip › Pfaff_et_al_2019_Supplemental_Information.pdf]

Supplementary Information for

Pfaff *et al.*: Proteogenomics uncovers critical elements of host response in bovine soft palate epithelial cells following *in vitro* infection with foot-and-mouth disease virus

Supplementary Figures

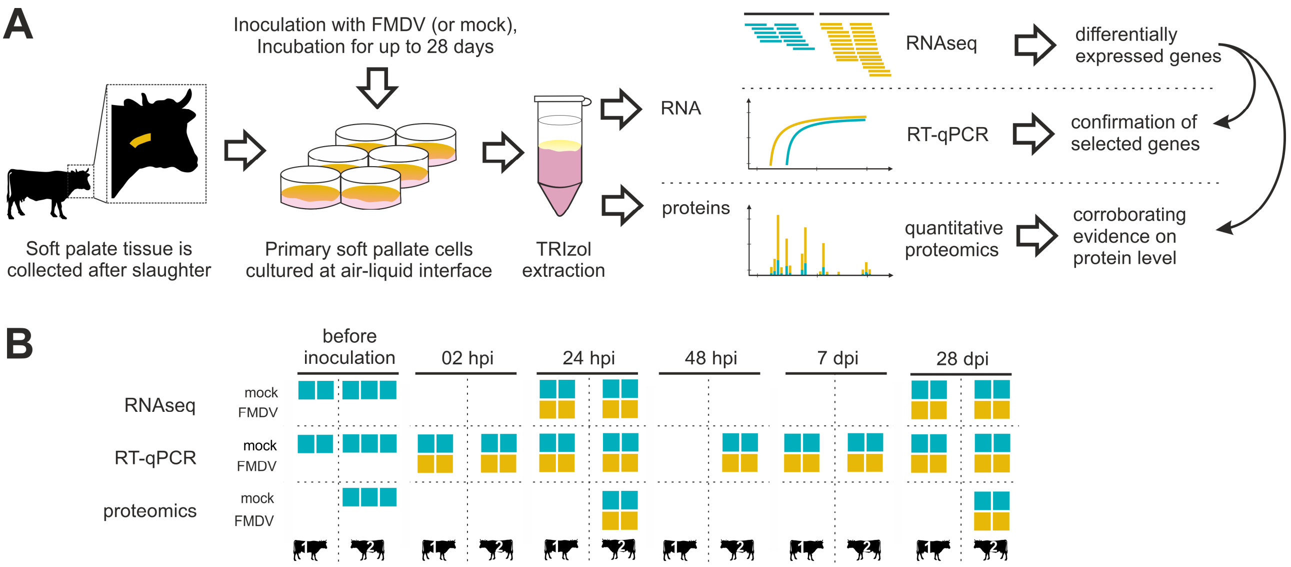

Figure S1. Schematic visualization of the proteogenomic workflow. (A). Soft palate (SP) tissue was received from two cattle that were slaughtered for food production. A primary SP cell culture was established at an air-liquid interface und was subsequently inoculated with lysate from infected (FMDV) or uninfected (mock) cell cultures. The inoculated SP cell cultures were then incubated for up to 28 days. At each sample time point, two replicate cultures were harvested and total RNA and proteins were isolated using TRIzol. The RNA fraction was used for comparative RNA sequencing and identification of differentially expressed genes. Among these, six target genes and two reference genes were selected for confirmation by RT-qPCR. Furthermore, the corresponding protein fraction was used for quantitative proteomics in order to corroborate the transcription data on the protein level. (B) Replicate SP cell cultures from the two animals (1 and 2, black silhouettes) were harvested at different time points: immediately before inoculation, 2 hours post inoculation (hpi), 24 hpi, 48 hpi, 7 days post inoculation (dpi) and 28 dpi. The heatmap shows the number of replicates (squares) from mock (blue) and FMDV infected (orange) cell cultures that have been analyzed using RNA sequencing, RT-qPCR or proteomics at each time point.

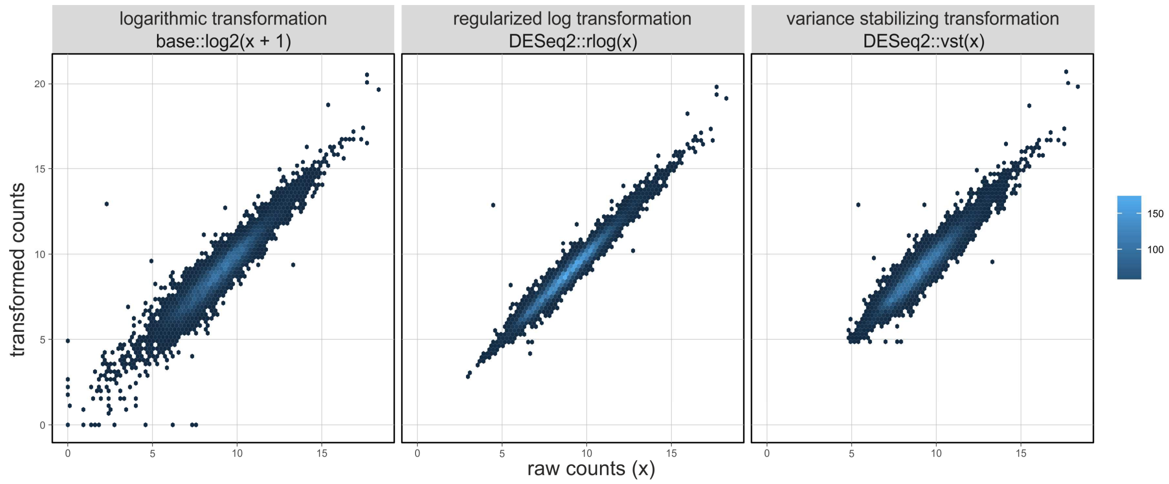

Figure S2. Comparison of different transformation methods for the raw reads counts per gene. The log2 transformation using sequence depth normalized counts (left) shows high deviation of transformed from raw counts that may artificially contribute to data variance. The regularized log (middle) and the variance stabilizing transformation as implemented in DESeq2 considers sequence depth and performs better than the logarithmic transformation. For variance-based approaches, the regularized log transformation was chosen as the best performing model. The color corresponds to the number of binned per gene counts and the function corresponds to the used R package and call.

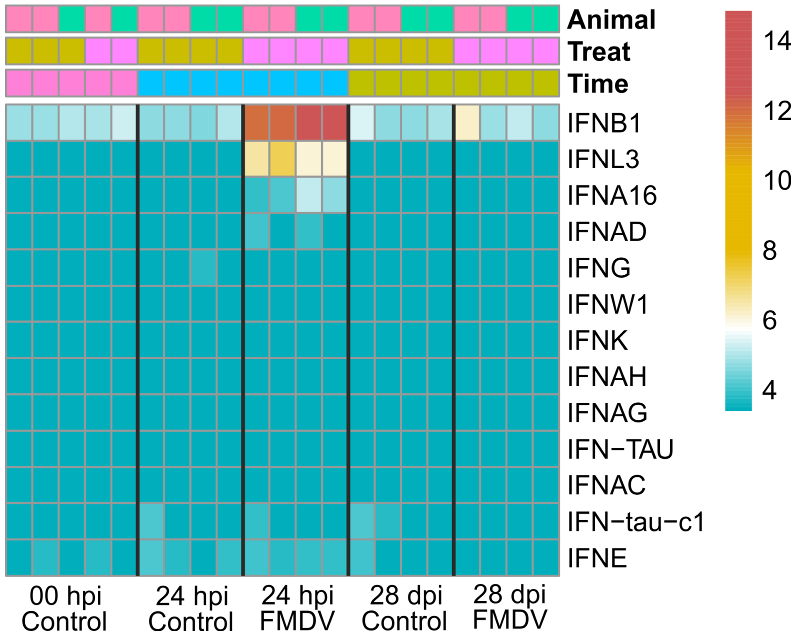

Figure S3. Comparison of interferon expression. The raw read counts for all available interferon typ I, II and III genes were extracted from an unfiltered dataset and transformed using the variance stabilizing transformation (vst) method, in order to avoid negative values for better visibility. The transformed data was visualized in a heatmap, were rows represent genes and columns represent samples from different time points (00 hpi, 24 hpi and 28 dpi), treatments (FMDV or controls) and animals.

Supplementary Tables

Table S1. Summary of primary soft palate cell culture samples used for RNA sequencing along with information on experimental treatment, such as inoculation agent and time as well as animal-specific properties and outcome of sequencing.

No	Sample ID	Treatment	Time	Animal	Total Reads
1	lib02459	control	0 hpi	1 (♀)	23,764,020
2	lib02460	control	0 hpi		23,830,051
3	lib02453	control	24 hpi		23,679,150
4	lib02454	control	24 hpi		25,959,245
5	lib02451	FMDV	24 hpi		24,194,883
6	lib02452	FMDV	24 hpi		26,512,041
7	lib02457	control	28 dpi		24,502,499
8	lib02458	control	28 dpi		24,923,161
9	lib02455	FMDV	28 dpi		22,879,371
10	lib02456	FMDV	28 dpi		25,383,683
11	lib02343	control	0 hpi	2 (♂)	23,766,199
12	lib02344	control	0 hpi		24,205,215
13	lib02360	control	0 hpi		25,499,001
14	lib02362	control	24 hpi		25,545,365
15	lib02346	control	24 hpi		24,372,618
16	lib02361	FMDV	24 hpi		25,894,882
17	lib02345	FMDV	24 hpi		24,405,453
18	lib02348	control	28 dpi		28,018,704
19	lib02350	control	28 dpi		24,381,295
20	lib02347	FMDV	28 dpi		21,855,393
21	lib02349	FMDV	28 dpi		24,818,395

Table S2. Summary of excluded and included RNA reference transcripts. A total of 57,846 RNA sequences of the *Bos taurus* reference genome (GCF000003055.6 Bos Taurus UMD 3.1.1) were filtered according to their RNA feature qualifiers and only a subset of reference sequences were further included in the analysis of differently expressed genes

	Qualifier	Entries	Description
**Included**			
	mRNA	48,655	messenger RNA
	partial mRNA	335	partial messenger RNA
	ncRNA	6169	non-coding RNA <200 nt
	non-coding RNA	25	non-coding RNA <200 nt
	long non-coding RNA	43	non-coding RNA >200 nt
	misc RNA	1808	any transcript or RNA product that cannot be defined by other RNA keys
	**total**	**57,035**	
**Excluded**			
	microRNA	806	micro RNA ~22 nt
	telomerase RNA	1	RNA component of telomerase
	ribosomal RNA	2	ribosomal ribonucleic acid
	RNase MRP RNA	1	RNA molecule essential for the catalytic activity of RNase MRP
	SRP RNA	1	signal recognition particle
	**total**	**811**	

S3 Table. Replaced gene aliases of the *Bos taurus* reference genome (GCF000003055.6 Bos Taurus UMD 3.1.1)

Original	Replacement	Description	Associated Accession
C28H10ORF10	DEPP1	Autophagy Regulator	NM_001046515.2
C10H15ORF48	NMES1	Normal mucosa of esophagus-specific gene 1 protein	NM_001206976.1
C7H19ORF66	C19ORF66	Repressor Of Yield Of Dengue Virus	NM_001038181.1
C1H21ORF91	C21ORF91	Cold Sore Susceptibility Gene 1	XM_005201150.3, XM_005201152.3, XM_010800942.2, NM_001037617.2
C1H3ORF38	C3ORF38	May be involved in apoptosis regulation	NM_001206451.2, XM_005201179.3
PVRL2	NECTIN2	Nectin Cell Adhesion Molecule 2	XM_005219196.1, XM_005219198.1
C3H1ORF109	C1ORF109	May promote cancer cell proliferation.	XM_010803595.2, NM_001101892.1
C8H9ORF91	TMEM268	Transmembrane Protein 268	XM_005210539.3, XM_005210540.3, XM_005210541.3, XM_015472690.1
C11H2ORF44	WDCP	WD Repeat And Coiled Coil Containing	XM_005213088.3, XM_015473568.1, XM_002691506.5
LOC784525, LOC525550, LOC100337470, IFNB3	IFNB1	Interferon beta 1	XM_002689540.4, XM_002689541.3, XM_002689537.4, NM_001114297.1

S4 Table. Sequences of primers used for the RT-qPCR analysis

Target	Forward Primer (3' → 5')	Reverse Primer (3' → 5')
ACTB	CTCTTCCAGCCTTCCTTCCT	GGGCAGTGATCTCTTTCTGC
ANKRD1	GACGAACTTCGGGTCAACAT	CTCTGCCTCTCGCTGTTTCT
CASP7	GAACAGATGGCAAGACAGCA	AACGGGGATTAGCATCAGTG
GAPDH	GGGTCATCATCTCTGCACCT	GGTCATAAGTCCCTCCACGA
IDO1	TGCTGTATGAAGGCATCTGG	TGAGCTGGTGGCATGTATGT
IFIH1	CGAAGTCAAAGAGCCACTCC	TGCCCATGTTGCTGTTATGT
NCAM1	CATCTACAACGCCAACATCG	CTTCTCCCTCCCTGAACTCC
OAS2	AACGAGTCACCTGGACCATC	GCTGGAGAAAGTCCTTGACG

S5 Table. Differentially expressed genes with an adjusted p-value (padj) <0.001 and a |log2 fold change| (LFC) >1

Gene	Entrez ID	Gene Description	24 hpi		28 dpi	
			LFC	padj	LFC	padj
**ABCD4**	5826	ATP binding cassette subfamily D member 4	3.06	1.83E-10		
**ABHD1**	84696	abhydrolase domain containing 1	5.72	6.33E-19		
**ACSL5**	51703	acyl-CoA synthetase long chain family member 5	2.05	1.36E-07		
**ADAR**	103	adenosine deaminase, RNA specific	2.76	2.24E-11		
**ADPGK**	83440	ADP dependent glucokinase	2.26	5.35E-05		
**AIDA**	64853	axin interactor, dorsalization associated	2.16	1.82E-08		
**ALDH1A2**	8854	aldehyde dehydrogenase 1 family member A2			-3.09	7.84E-05
**ANKRD1**	27063	ankyrin repeat domain 1			-4.19	6.58E-05
**ANXA1**	301	annexin A1	2.79	1.70E-13		
**APOBEC3B**	9582	apolipoprotein B mRNA editing enzyme catalytic subunit 3B	2.88	2.80E-06		
**AREL1**	9870	apoptosis resistant E3 ubiquitin protein ligase 1	1.79	2.74E-06		
**ARF3**	377	ADP ribosylation factor 3	2.17	1.77E-04		
**ARNTL2**	56938	aryl hydrocarbon receptor nuclear translocator like 2	3.30	3.11E-05		
**ARSH**	347527	arylsulfatase family member H	2.77	1.26E-07		
**ATAD1**	84896	ATPase family, AAA domain containing 1	2.59	5.03E-07		
**ATP10A**	57194	ATPase phospholipid transporting 10A (putative)	2.91	5.49E-07		
**ATP8B4**	79895	ATPase phospholipid transporting 8B4 (putative)	3.60	1.08E-25		
**ATXN3**	4287	ataxin 3	2.63	1.20E-05		
**BAG3**	9531	BCL2 associated athanogene 3	1.97	4.79E-05		
**BATF2**	116071	basic leucine zipper ATF-like transcription factor 2	4.83	9.27E-56		
**BCL2L12**	83596	BCL2 like 12	3.07	5.71E-13		
**BHLHE41**	79365	basic helix-loop-helix family member e41	3.48	1.36E-04		
**BIN3**	55909	bridging integrator 3	2.00	2.56E-06		
**BOLA-DMB**	282491	major histocompatibility complex, class II, DM beta	3.63	1.41E-17		
**BPNT1**	10380	3'(2'), 5'-bisphosphate nucleotidase 1	3,25	3.02E-13		
**C19ORF66**			3.14	7.30E-08		
**C1ORF109**			2.25	6.69E-08		
**C21ORF91**			3.01	2.38E-05		
**C3ORF38**			2.70	4.88E-11		
**CA13**	377677	carbonic anhydrase 13	-3.22	3.56E-04		
**CALCOCO2**	10241	calcium binding and coiled-coil domain 2	2.58	1.65E-08		
**CASP4**	837	caspase 4	2.98	1.07E-06		
**CASP7**	840	caspase 7	2.66	2.59E-13		
**CCDC82**	79780	coiled-coil domain containing 82	1.92	4.37E-05		
**CCL2**	6347	C-C motif chemokine ligand 2	3.17	3.24E-10		
**CCL5**	6352	C-C motif chemokine ligand 5	6.66	8.74E-19	4.70	3.02E-07
**CCL8**	6355	C-C motif chemokine ligand 8	11.79	1.00E-21		
**CD200**	4345	CD200 molecule	3.30	1.19E-05		
**CD274**	29126	CD274 molecule	4.67	2.71E-25		
**CD40**	958	CD40 molecule	4.29	2.84E-13		

**CD83**	9308	CD83 molecule	5.08	8.81E-05		
**CDADC1**	81602	cytidine and dCMP deaminase domain containing 1	3.40	1.25E-33		
**CDKN2AIP**	55602	CDKN2A interacting protein	3.19	1.10E-18		
**CELA3B**	23436	chymotrypsin like elastase family member 3B	2.78	1.54E-04		
**CFLAR**	8837	CASP8 and FADD like apoptosis regulator	2.88	2.79E-05		
**CHST12**	55501	carbohydrate sulfotransferase 12	1.88	1.19E-04		
**CLEC2D**	29121	C-type lectin domain family 2 member D	3.45	7.80E-08		
**CMPK2**	129607	cytidine/uridine monophosphate kinase 2	8.22	6.07E-39	4.37	8.48E-08
**CMTR2**	55783	cap methyltransferase 2	3.48	3.24E-20		
**CNP**	1267	2',3'-cyclic nucleotide 3' phosphodiesterase	2.25	2.32E-10		
**COL1A1**	1277	collagen type I alpha 1 chain			-3.50	4.15E-04
**COL3A1**	1281	collagen type III alpha 1 chain			-3.28	3.22E-04
**CRYM**	1428	crystallin mu	5.73	3.78E-08		
**CSDC2**	27254	cold shock domain containing C2	3.86	3.21E-28		
**CSRNP1**	64651	cysteine and serine rich nuclear protein 1	2.64	2.49E-04		
**CTC1**	80169	CST telomere replication complex component 1	3.26	3.61E-16		
**CTSS**	1520	cathepsin S	2.84	8.90E-08		
**CXCL10**	3627	C-X-C motif chemokine ligand 10	11.01	1.33E-12		
**CXCL2**	2920	C-X-C motif chemokine ligand 2	6.08	4.82E-06		
**CXCL8**	3576	C-X-C motif chemokine ligand 8	6.68	3.76E-05		
**CYP2J2**	1573	cytochrome P450 family 2 subfamily J member 2	7.05	1.28E-07		
**CYTH1**	9267	cytohesin 1	1.98	2.52E-06		
**DAXX**	1616	death domain associated protein	2.82	1.21E-13		
**DDX58**	23586	DExD/H-box helicase 58	5.18	4.24E-77	3.72	1.08E-31
**DEPP1**	11067	DEPP1, autophagy regulator	3.61	6.41E-05		
**DHX58**	79132	DExH-box helicase 58	5.86	2.92E-75	3.99	2.90E-27
**DNAJC1**	64215	DnaJ heat shock protein family (Hsp40) member C1	2.42	4.92E-07		
**DRAM1**	55332	DNA damage regulated autophagy modulator 1	3.49	1.21E-24		
**DTX3L**	151636	deltex E3 ubiquitin ligase 3L	4.82	6.81E-94	3.61	2.86E-42
**EGR2**	1959	early growth response 2	3.67	2.65E-17		
**EHD4**	30844	EH domain containing 4	3.46	1.21E-09		
**EIF2AK2**	5610	eukaryotic translation initiation factor 2 alpha kinase 2	3.33	2.23E-08		
**ELMO2**	63916	engulfment and cell motility 2	2.36	2.84E-13		
**EPSTI1**	94240	epithelial stromal interaction 1	4.51	1.86E-15	3.32	4.47E-06
**ERAP2**	64167	endoplasmic reticulum aminopeptidase 2	2.29	8.35E-05		
**ESPNL**	339768	espin like	2.78	2.31E-05		
**FAM111B**	374393	family with sequence similarity 111 member B	3.08	2.21E-04		
**FAM192A**	80011	family with sequence similarity 192 member A	2.20	1.24E-05		
**FAM3B**	54097	family with sequence similarity 3 member B	9.41	8.65E-13	6.80	2.65E-05
**FAM46A**	55603	family with sequence similarity 46 member A	2.51	2.24E-07		
**FBP1**	2203	fructose-bisphosphatase 1			4.09	2.93E-05
**FBXO16**	157574	F-box protein 16	3.17	3.24E-09		
**FBXO33**	254170	F-box protein 33	3.61	6.56E-13		

**FCHSD2**	9873	FCH and double SH3 domains 2	2.40	1.44E-04		
**FITM2**	128486	fat storage inducing transmembrane protein 2	-2.33	3.75E-10		
**FMDV**		Foot-and-mouth disease virus genome	17.76	6.46E-35		
**FOXS1**	2307	forkhead box S1	4.80	4.12E-05		
**GBP2**	2634	guanylate binding protein 2	6.25	2.03E-44	3.16	8.70E-07
**GBP4**	115361	guanylate binding protein 4	9.44	8.43E-36	7.58	2.83E-21
**GBP5**	115362	guanylate binding protein 5	6.39	1.50E-30	3.57	3.88E-06
**GMIP**	51291	GEM interacting protein	2.84	2.52E-08		
**GPR63**	81491	G protein-coupled receptor 63	3.41	2.35E-04		
**GPR65**	8477	G protein-coupled receptor 65	2.70	5.10E-04		
**GRINA**	2907	glutamate ionotropic receptor NMDA type subunit associated protein 1	2.64	4.05E-13		
**GTF2B**	2959	general transcription factor IIB	4.44	1.31E-40		
**HBEGF**	1839	heparin binding EGF like growth factor	3.63	2.88E-10		
**HECTD2**	143279	HECT domain E3 ubiquitin protein ligase 2	3.96	2.16E-17		
**HERC5**	51191	HECT and RLD domain containing E3 ubiquitin protein ligase 5	4.83	2.25E-23	2.77	2.57E-04
**HERC6**	55008	HECT and RLD domain containing E3 ubiquitin protein ligase family member 6	5.42	3.58E-48	3.87	9.44E-20
**HOMEZ**	57594	homeobox and leucine zipper encoding	2.11	2.49E-11		
**HS3ST1**	9957	heparan sulfate-glucosamine 3-sulfotransferase 1	4.31	4.36E-09		
**HTRA3**	94031	HtrA serine peptidase 3			-3.21	2.93E-07
**IDO1**	3620	indoleamine 2,3-dioxygenase 1	11.56	2.47E-47		
**IER2**	9592	immediate early response 2	2.17	7.38E-05		
**IFI16**	3428	interferon gamma inducible protein 16	3.84	5.81E-19	2.53	7.33E-05
**IFI27**	3429	interferon alpha inducible protein 27	8.11	2.71E-101	8.18	1.95E-92
**IFI35**	3430	interferon induced protein 35	3.40	1.05E-19	2.31	4.96E-05
**IFI44**	10561	interferon induced protein 44	6.70	2.33E-20	5.52	3.24E-12
**IFI44L**	10964	interferon induced protein 44 like	5.74	7.25E-67	3.89	2.65E-24
**IFI6**	2537	interferon alpha inducible protein 6	6.00	8.16E-20	5.85	3.98E-18
**IFIH1**	64135	interferon induced with helicase C domain 1	6.49	9.33E-60	4.30	5.93E-21
**IFIT2**	3433	interferon induced protein with tetratricopeptide repeats 2	10.98	7.92E-89	5.94	5.93E-21
**IFIT3**	3437	interferon induced protein with tetratricopeptide repeats 3	6.74	1.38E-22		
**IFIT5**	24138	interferon induced protein with tetratricopeptide repeats 5	2.83	5.12E-13		
**IFITM1**	8519	interferon induced transmembrane protein 1	4.12	4.96E-19	3.58	2.05E-12
**IFITM2**	10581	interferon induced transmembrane protein 2	9.76	1.90E-36		
**IFNB1**	3456	interferon beta 1	10.47	5.67E-53		
**IL15RA**	3601	interleukin 15 receptor subunit alpha	4.04	1.76E-15		
**IL18**	3606	interleukin 18	2.95	1.05E-12		
**IL18BP**	10068	interleukin 18 binding protein	2.42	2.05E-07		
**IL3RA**	3563	interleukin 3 receptor subunit alpha	6.19	2.65E-17		
**IL4I1**	259307	interleukin 4 induced 1	4.04	5.43E-13		
**IL6**	3569	interleukin 6	6.21	2.15E-09		
**IP6K1**	9807	inositol hexakisphosphate kinase 1	2.10	5.69E-10		
**IQCD**	115811	IQ motif containing D	3.81	1.82E-08		
**IRF1**	3659	interferon regulatory factor 1	3.60	4.93E-07		

**IRF3**	3661	interferon regulatory factor 3	2.06	7.33E-05		
**IRF5**	3663	interferon regulatory factor 5	3.46	4.08E-04		
**IRF7**	3665	interferon regulatory factor 7	4.99	9.68E-22	2.85	4.52E-04
**IRF9**	10379	interferon regulatory factor 9	3.10	3.76E-32	1.99	2.61E-06
**ISG15**	9636	ISG15 ubiquitin-like modifier	11.18	3.46E-80	8.99	8.10E-47
**ITPR2**	3709	inositol 1,4,5-trisphosphate receptor type 2	2.32	6.00E-14		
**JAKMIP3**	282973	Janus kinase and microtubule interacting protein 3	4.23	5.42E-22		
**KIF5C**	3800	kinesin family member 5C	9.89	5.28E-32		
**KIFC3**	3801	kinesin family member C3	2.73	3.48E-04		
**LAYN**	143903	layilin	3.38	4.51E-06		
**LEKR1**	389170	leucine, glutamate and lysine rich 1	4.93	4.45E-20		
**LGALS3BP**	3959	galectin 3 binding protein	2.52	1.63E-04		
**LGALS9**	3965	galectin 9	4.37	1.06E-04		
**LOC100139670**	100139670	interferon-induced protein with tetratricopeptide repeats 1	5.81	6.09E-21	3.58	2.32E-05
**LOC100298356**	100298356	bone marrow stromal antigen 2	9.04	7.68E-34	6.48	4.52E-15
**LOC100336669**	100336669	guanylate-binding protein 4	5.96	1.91E-50	3.25	1.25E-09
**LOC101902757**	101902757	uncharacterized LOC101902757	4.32	4.04E-05		
**LOC101903126**	101903126	interferon-induced very large GTPase 1-like	5.94	3.92E-04		
**LOC101903326**	101903326	zinc finger protein 485	-2.20	5.86E-04		
**LOC101903473**	101903473	uncharacterized LOC101903473	4.07	9.72E-24	2.43	3.46E-04
**LOC101905355**	101905355	uncharacterized LOC101905355	5.49	3.05E-11		
**LOC101907348**	101907348	uncharacterized LOC101907348	3.47	1.61E-09		
**LOC101907799**	101907799	uncharacterized LOC101907799	13.48	2.47E-29	8.67	3.94E-11
**LOC101907813**	101907813	uncharacterized LOC101907813	3.39	3.77E-08		
**LOC101908206**	101908206	uncharacterized LOC101908206	-2.64	8.82E-05		
**LOC104968925**	104968925	uncharacterized LOC104968925	5.14	1.55E-25		
**LOC104970162**	104970162	uncharacterized LOC104970162	3.31	1.48E-07		
**LOC104970840**	104970840	uncharacterized LOC104970840	4.02	5.55E-10		
**LOC104971726**	104971726	uncharacterized LOC104971726	7.96	8.45E-16		
**LOC104972593**	104972593	uncharacterized LOC104972593	10.67	1.83E-16	7.17	5.19E-06
**LOC104973892**	104973892	uncharacterized LOC104973892	3.51	5.51E-15		
**LOC104975716**	104975716	leukocyte elastase inhibitor pseudogene	2.23	6.64E-05		
**LOC107131142**	107131142	tumor necrosis factor ligand superfamily member 13B-like	5.70	8.21E-08	4.79	2.57E-04
**LOC107131199**	107131199	cysteine/serine-rich nuclear protein 2	2.14	4.04E-04		
**LOC107131472**	107131472	vomeronasal type-1 receptor 4-like	3.20	1.20E-05		
**LOC107131864**	107131864	eukaryotic translation initiation factor 4E pseudogene	4.91	1.77E-17	3.04	3.87E-04
**LOC107132217**	107132217	uncharacterized LOC107132217	8.54	1.06E-29	6.48	1.59E-14
**LOC107132617**	107132617	uncharacterized LOC107132617	3.97	7.61E-09		
**LOC107132911**	107132911	uncharacterized LOC107132911	2.32	2.84E-04		
**LOC507055**	507055	guanylate-binding protein 4	7.60	1.28E-73	4.54	2.18E-20
**LOC508153**	508153	uncharacterized LOC508153	3.47	1.54E-07	2.96	8.43E-04
**LOC509283**	509283	E3 ubiquitin-protein ligase RNF213	4.76	1.55E-80	2.87	2.50E-19
**LOC510382**	510382	guanylate-binding protein 6-like	6.29	1.08E-54	3.19	1.01E-08
**LOC511531**	511531	guanylate-binding protein 1	5.70	1.00E-38	2.63	5.14E-04

**LOC511936**	511936	cytochrome P450, family 2, subfamily J	6.32	4.29E-06		
**LOC512486**	512486	interferon-induced guanylate-binding protein 1	5.51	2.37E-42	4.13	3.23E-19
**LOC512672**	512672	major histocompatibility complex, class I	2.44	6.07E-09		
**LOC513659**	513659	guanylate-binding protein 6	7.06	2.17E-10		
**LOC613699**	613699	interferon-induced transmembrane protein 1	3.42	4.20E-05		
**LOC616948**	616948	tripartite motif-containing protein 5-like	3.41	1.65E-12		
**LOC618409**	618409	interferon regulatory factor 4	7.12	1.45E-49	3.40	3.07E-07
**LOC618737**	618737	bone marrow stromal antigen 2	10.33	8.38E-119	8.70	7.29E-46
**LOC781710**	781710	guanylate-binding protein 2	7.78	1.45E-49	6.10	9.04E-20
**LOC783920**	783920	interferon-induced very large GTPase 1-like	5.14	3.14E-14		
**LOC786073**	786073	interferon-induced transmembrane protein 1-like	4.04	5.67E-04		
**LOC786372**	786372	golgin subfamily A member 6-like protein 22	5.33	2.84E-18		
**LY6E**	4061	lymphocyte antigen 6 family member E			3.45	2.61E-06
**MAD2L2**	10459	mitotic arrest deficient 2 like 2	3.14	3.33E-14		
**MANF**	7873	mesencephalic astrocyte derived neurotrophic factor	2.25	2.84E-04		
**MAP1LC3C**	440738	microtubule associated protein 1 light chain 3 gamma	7.76	1.03E-05		
**MAP3K8**	1326	mitogen-activated protein kinase kinase kinase 8	2.31	4.65E-04		
**MARCKSL1**	65108	MARCKS like 1	3.00	1.65E-08		
**MASTL**	84930	microtubule associated serine/threonine kinase like	3.21	9.94E-10		
**MAT2B**	27430	methionine adenosyltransferase 2B	2.00	5.83E-04		
**MB21D1**	782472	Mab-21 domain containing 1	6.72	5.14E-38	3.32	1.60E-05
**MCHR1**	2847	melanin concentrating hormone receptor 1	6.14	3.85E-22		
**MED17**	9440	mediator complex subunit 17	2.12	2.89E-07		
**MIC1**	533051	major histocompatibility class I related protein	3.41	2.31E-15		
**MITD1**	129531	microtubule interacting and trafficking domain containing 1	2.39	5.63E-06		
**MOV10**	4343	Mov10 RISC complex RNA helicase	2.98	6.40E-08		
**MSC**	9242	musculin	2.03	2.05E-04		
**MTFP1**	51537	mitochondrial fission process 1	2.35	1.85E-05		
**MUC5B**	727897	mucin 5B, oligomeric mucus/gel-forming	4.71	1.41E-11		
**MX1**	4599	MX dynamin like GTPase 1	8.04	4.06E-164	6.87	4.53E-112
**MX2**	4600	MX dynamin like GTPase 2	13.02	3.92E-122	10.00	1.56E-66
**MYLK**	4638	myosin light chain kinase			-3.26	9.60E-07
**NAMPT**	10135	nicotinamide phosphoribosyltransferase	2.99	5.70E-06		
**NCAM1**	4684	neural cell adhesion molecule 1			-4.48	3.34E-04
**NECTIN2**	5819	nectin cell adhesion molecule 2	2.40	6.67E-12		
**NFKBIA**	4792	NFKB inhibitor alpha	3.75	1.02E-07		
**NFKBIB**	4793	NFKB inhibitor beta	2.17	1.29E-05		
**NFKBIE**	4794	NFKB inhibitor epsilon	2.70	4.20E-05		
**NLRC5**	84166	NLR family CARD domain containing 5	4.40	1.17E-07		
**NMES1**	84419	Normal Mucosa Of Esophagus Specific 1	3.43	3.23E-23		
**NOTCH3**	4854	notch 3	3.68	5.25E-05		
**NR3C1**	2908	nuclear receptor subfamily 3 group C member 1	2.09	9.67E-09		

**NR4A3**	8013	nuclear receptor subfamily 4 group A member 3	4.71	4.15E-08		
**NUB1**	51667	negative regulator of ubiquitin like proteins 1	2.01	2.04E-04		
**OAS1X**	347699	2',5'-oligoadenylate synthetase 1, 40/46kDa	6.41	3.86E-81	5.82	1.47E-60
**OAS1Y**	654488	2',5'-oligoadenylate synthetase 1, 40/46kDa	6.70	1.35E-61	5.84	1.77E-43
**OAS1Z**	519922	2',5'-oligoadenylate synthetase 1, 40/46kDa	6.99	9.33E-60	6.78	2.30E-49
**OAS2**	4939	2'-5'-oligoadenylate synthetase 2	9.15	5.93E-80	6.01	2.45E-28
**OLR1**	4973	oxidized low density lipoprotein receptor 1	4.20	5.44E-08		
**ONECUT2**	9480	one cut homeobox 2	4.03	4.78E-04		
**PARM1**	25849	prostate androgen-regulated mucin-like protein 1	4.76	1.93E-11		
**PARP10**	84875	poly(ADP-ribose) polymerase family member 10	4.30	7.31E-19		
**PARP12**	64761	poly(ADP-ribose) polymerase family member 12	4.65	2.49E-40	3.32	1.35E-15
**PARP14**	54625	poly(ADP-ribose) polymerase family member 14	4.51	1.34E-55	3.03	4.57E-18
**PARP9**	83666	poly(ADP-ribose) polymerase family member 9	3.53	7.94E-43	3.44	1.50E-38
**PDE12**	201626	phosphodiesterase 12	1.90	9.11E-05		
**PDK1**	5163	pyruvate dehydrogenase kinase 1	-2.43	7.00E-05		
**PELI1**	57162	pellino E3 ubiquitin protein ligase 1	1.97	7.84E-04		
**PHLDA1**	22822	pleckstrin homology like domain family A member 1	3.39	5.96E-05		
**PLAC8**	51316	placenta specific 8	8.34	1.87E-14	8.80	4.74E-11
**PLEKHA4**	57664	pleckstrin homology domain containing A4	3.50	8.77E-36		
**PLSCR2**	57047	phospholipid scramblase 2	3.43	1.93E-11		
**PMAIP1**	5366	phorbol-12-myristate-13-acetate-induced protein 1	2.69	1.31E-08		
**PML**	5371	promyelocytic leukemia	4.11	1.53E-30		
**PNPT1**	87178	polyribonucleotide nucleotidyltransferase 1	3.65	2.83E-20		
**POLH**	5429	DNA polymerase eta	2.46	2.07E-07		
**PPA1**	5464	pyrophosphatase (inorganic) 1	2.23	1.55E-05		
**PPP1R15A**	23645	protein phosphatase 1 regulatory subunit 15A	2.98	3.88E-11		
**PPP2R3C**	55012	protein phosphatase 2 regulatory subunit B''gamma	3.46	8.19E-13		
**PRICKLE3**	4007	prickle planar cell polarity protein 3	2.72	2.84E-08		
**PSMB10**	5699	proteasome subunit beta 10	2.41	2.18E-04		
**PSMB8**	5696	proteasome subunit beta 8	3.30	2.94E-09		
**PSMB9**	5698	proteasome subunit beta 9	4.31	2.05E-06		
**PSME2**	5721	proteasome activator subunit 2	2.22	2.29E-04		
**PSMF1**	9491	proteasome inhibitor subunit 1	2.87	7.13E-07		
**PTGIR**	5739	prostaglandin I2 receptor	5.60	1.56E-05		
**RAB37**	326624	RAB37, member RAS oncogene family	5.82	3.56E-04		
**RAB3IL1**	5866	RAB3A interacting protein like 1	-1.91	9.57E-04		
**RAB8B**	51762	RAB8B, member RAS oncogene family	2.45	3.39E-06		
**RASSF1**	11186	Ras association domain family member 1	2.96	1.32E-10		
**RBCK1**	10616	RANBP2-type and C3HC4-type zinc finger containing 1	1.82	3.60E-04		
**RBM43**	375287	RNA binding motif protein 43	2.95	4.71E-04		
**RBMS2**	5939	RNA binding motif single stranded interacting protein 2	2.42	3.38E-06		

**RHBDF2**	79651	rhomboid 5 homolog 2	2.64	2.36E-04		
**RIPK3**	11035	receptor interacting serine/threonine kinase 3	2.64	4.33E-06		
**RNASEL**	6041	ribonuclease L	2.54	2.33E-06		
**RNF114**	55905	ring finger protein 114	2.48	4.07E-06		
**RNF19B**	127544	ring finger protein 19B	3.30	2.92E-13		
**RSAD2**	91543	radical S-adenosyl methionine domain containing 2	12.17	1.59E-81	9.44	4.70E-42
**RSL1D1**	26156	ribosomal L1 domain containing 1	1.92	1.03E-09		
**RTP4**	64108	receptor transporter protein 4	4.15	5.96E-30	2.47	8.65E-06
**SALL2**	6297	spalt like transcription factor 2	-3.44	7.68E-06		
**SAMD9**	54809	sterile alpha motif domain containing 9	4.50	5.85E-08		
**SAT1**	6303	spermidine/spermine N1-acetyltransferase 1	2.50	2.07E-04		
**SCLY**	51540	selenocysteine lyase	3.47	2.62E-12		
**SCRN1**	9805	secernin 1	-3.68	1.07E-05		
**SFRP2**	6423	secreted frizzled related protein 2			-4.16	3.30E-05
**SLC15A3**	51296	solute carrier family 15 member 3	6.42	2.05E-45		
**SLC25A19**	60386	solute carrier family 25 member 19	3.23	3.66E-32		
**SLC25A28**	81894	solute carrier family 25 member 28	2.37	4.49E-05		
**SLFN11**	91607	schlafen family member 11	6.08	4.79E-21	3.71	2.64E-05
**SLFN12**	55106	schlafen family member 12	3.30	1.03E-05		
**SMAGP**	57228	small cell adhesion glycoprotein	2.44	6.06E-05		
**SOCS1**	8651	suppressor of cytokine signaling 1	3.64	7.36E-05		
**SP100**	6672	SP100 nuclear antigen	4.95	1.30E-21	3.07	3.31E-05
**SP110**	3431	SP110 nuclear body protein	4.49	4.04E-40		
**SP140**	11262	SP140 nuclear body protein	4.06	3.53E-10		
**SP140L**	93349	SP140 nuclear body protein like	2.38	2.03E-07		
**SPTLC2**	9517	serine palmitoyltransferase long chain base subunit 2	2.08	5.88E-05		
**STARD5**	80765	StAR related lipid transfer domain containing 5	1.94	1.62E-04		
**STARD8**	9754	StAR related lipid transfer domain containing 8	2.72	5.98E-09		
**STAT2**	6773	signal transducer and activator of transcription 2	2.42	1.47E-11		
**STK17A**	9263	serine/threonine kinase 17a	2.66	7.14E-05		
**SVIL**	6840	supervillin	2.13	7.22E-06		
**SYNM**	23336	synemin	2.37	7.00E-05		
**SYTL3**	94120	synaptotagmin like 3	4.13	1.08E-10		
**TAF3**	83860	TATA-box binding protein associated factor 3	2.46	1.08E-05		
**TAF4B**	6875	TATA-box binding protein associated factor 4b	3.62	6.19E-17		
**TAP1**	6890	transporter 1, ATP binding cassette subfamily B member	4.46	5.65E-04		
**TDRD7**	23424	tudor domain containing 7	2.91	4.02E-24		
**TIFA**	92610	TRAF interacting protein with forkhead associated domain	5.63	6.96E-17		
**TMEM106A**	113277	transmembrane protein 106A	3.61	1.05E-24		
**TMEM140**	55281	transmembrane protein 140	2.96	2.88E-10		
**TMEM268**	203197	transmembrane protein 268	2.07	4.79E-04		
**TMPRSS2**	7113	transmembrane protease, serine 2	3.08	2.52E-04		
**TNFAIP1**	7126	TNF alpha induced protein 1	1.95	9.12E-04		

**TNFAIP3**	7128	TNF alpha induced protein 3	4.58	3.34E-17		
**TNFSF10**	8743	TNF superfamily member 10	6.44	2.09E-32	3.79	2.22E-08
**TNFSF4**	7292	TNF superfamily member 4	3.48	3.76E-10		
**TRANK1**	9881	tetratricopeptide repeat and ankyrin repeat containing 1	3.37	2.09E-23		
**TREX1**	11277	three prime repair exonuclease 1	3.80	2.81E-26		
**TRIM14**	9830	tripartite motif containing 14	3.83	2.26E-04		
**TRIM16**	10626	tripartite motif containing 16	2.33	1.63E-09		
**TRIM21**	6737	tripartite motif containing 21	4.10	2.79E-34		
**TRIM25**	7706	tripartite motif containing 25	2.29	9.35E-14		
**TRIM34**	53840	tripartite motif containing 34	3.77	1.94E-10		
**TRIM38**	10475	tripartite motif containing 38	3.01	9.76E-16		
**TRIM56**	81844	tripartite motif containing 56	1.98	4.54E-05		
**TSKU**	25987	tsukushi, small leucine rich proteoglycan	2.51	3.14E-07		
**TWISTNB**	221830	TWIST neighbor	1.94	1.46E-04		
**UBA7**	7318	ubiquitin like modifier activating enzyme 7	5.31	3.66E-24	3.64	2.33E-08
**UNC93B1**	81622	unc-93 homolog B1, TLR signaling regulator	1.91	8.06E-06		
**USP18**	11274	ubiquitin specific peptidase 18	6.91	4.84E-61	5.00	8.63E-27
**USP25**	29761	ubiquitin specific peptidase 25	2.35	2.58E-06		
**WARS**	7453	tryptophanyl-tRNA synthetase	3.24	4.42E-04		
**WDCP**	80304	WD repeat and coiled coil containing	1.96	5.89E-05		
**XAF1**	54739	XIAP associated factor 1	7.06	2.28E-61	5.63	1.79E-35
**XRN2**	22803	5'-3' exoribonuclease 2	1.96	2.79E-05		
**ZBP1**	81030	Z-DNA binding protein 1	8.48	7.27E-122	7.17	2.65E-70
**ZCCHC18**	644353	zinc finger CCHC-type containing 18	2.56	2.18E-04		
**ZMYM5**	9205	zinc finger MYM-type containing 5	2.16	4.62E-04		
**ZNFX1**	57169	zinc finger NFX1-type containing 1	4.15	9.33E-60	2.76	4.05E-18
