# Supplementary figures and images for: Proteogenomics Uncovers Critical Elements of Host Response in Bovine Soft Palate Epithelial Cells Following In Vitro Infection with Foot-And-Mouth Disease Virus
Source: Viruses. 2019 Jan 12;11(1):53. doi: 10.3390/v11010053 (PMC6356718; doi:10.3390/v11010053)

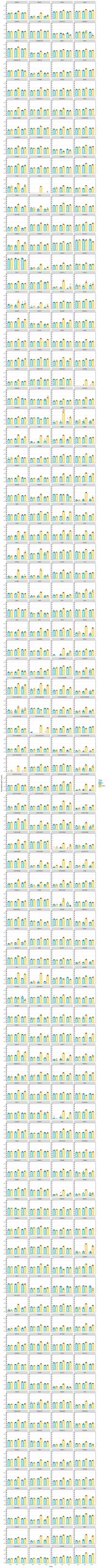

Supplement: Supplementary file 1 [file viruses-11-00053-s001.zip › Pfaff_et_al_2019_Supplemental_File_S1.pdf]
